# LoRaWAN Behaviour Analysis through Dataset Traffic Investigation

**DOI:** 10.3390/s22072470

**Published:** 2022-03-23

**Authors:** Pietro Spadaccino, Francesco Giuseppe Crinó, Francesca Cuomo

**Affiliations:** 1 DIET, Department of Information Engineering, Electronics and Telecommunications, Sapienza University of Rome, 00184 Rome, Italy; francesca.cuomo@uniroma1.it; 2 CNIT, Consorzio Nazionale Interuniversitario per le Telecomunicazioni, 00133 Parma, Italy; 3 Department of Computer, Control and Management Engineering, Sapienza University of Rome, 00185 Rome, Italy; crino.1954737@studenti.uniroma1.it

**Keywords:** LPWAN, LoRA, LoRaWAN, performance analysis

## Abstract

The large development of Internet of Things technologies is increasing the use of smart-devices to solve and support several real-life issues. In many cases, the aim is to move toward systems that, even if significant demands are not required in terms of quantity of exchanged data, they should be very reliable in terms of battery life and signal coverage. Networks that have these characteristics are the Low Power WAN (LPWAN). One of the most interesting LPWAN is LoRaWAN. LoRaWAN is a network with four principal components: end-devices, gateways, network servers, and application servers. It uses a LoRa physical layer to exchange messages between end-devices and gateways that forward these messages, through classic TCP/IP protocol, to the network server. In this work, we analyse LoRa and LoRaWAN by looking at its transmission characteristics and network behaviour, respectively, explaining the role of its components and showing the message exchange. This analysis is performed through the exploration of a dataset taken from the literature collecting real LoRaWAN packets. The goal of the work is twofold: (1) to investigate, under different perspectives, how a LoRaWAN works and (2) to provide software tools that can be used in several other LoraWAN datasets to measure the network behaviour. We carry out six different analyses to look at the most important features of LoRaWAN. For each analysis we present the adopted measurement strategy as well as the obtained results in the specific use case.

## 1. Introduction

The market for the Internet of Things (IoT) is becoming more and more appealing thanks to the multiplicity of applications and the suitable technologies available nowadays. In 2021, IoT Analytics expected the global number of connected IoT devices to grow 9%, to 12.3 billion active endpoints. By 2025, there will likely be more than 27 billion IoT connections [1].

IoT networks are more and more used to face aspects, such as security, smart industries, smart homes, utilities support, and many others. When using smart devices the most common issues are related to power consumption and signal coverage, for example in a smart city we do not want to deploy many access points around the city that require a big amount of resources in terms of power and maintenance. For that reason, the so called Low-Power Wide-Area Network (LPWAN) are quickly developing. These are networks that have features such as long-range signal coverage, low power consumption and low cost for the devices and their maintenance. To reach these goals it is necessary to use lightweight protocols to exchange data in order to reduce hardware and software complexity and power consumption.

In this market, there are already several LPWAN solutions, such as NB-IoT, which follow 3GPP standards and operate on licensed bands. Additionally, unlicensed solutions, out of 3GPP standards, which operate on the industrial, scientific, and medical (ISM) radio bands are available, such as Sig-Fox and LoRaWAN (Low Power Wide Area Network). LoRaWAN is one of the most interesting since with a powerful and robust physical layer, named LoRa, it is possible to set up long range communication links.

The contributions of the paper can be summarized in three main points. These are the following:

1. We aim at defining and using some performance metrics useful to evaluate LoRaWAN, as a network. As far as we know the contribution is new since few works have provided a way to process real LoRaWAN datasets to derive performance indicators. The most of the proposals in the literature are based on simulations while we have developed software tools to apply the metrics on any dataset;
2. We designed and presented the procedures implemented in our software tool that can be used to analyse real-world LoRaWAN data;
3. We tested these procedures on a large and heterogeneous dataset that contain data collected from a big LoRaWAN ecosystem with 9 gateways and used for several distinct IoT applications.

The rest of the paper is organized as following. We provide a quick overview of LoRa and LoRaWAN in Section 2. Then in Section 3 we presented the LoRaWAN at the Edge Dataset (LoED) that contains a rich collection of LoRaWAN packets gathered from a real LoRaWAN in London [2]. We use LoED as a study case to perform some data analyses. We present our six analyses in Section 4, for each analysis we introduce the type of measures we target to, the adopted strategy and we discuss the obtained results.

## 2. LoRaWAN in a Nutshell

LoRaWAN is a low-power, wide area networking protocol built on top of the LoRa radio modulation technique. It wirelessly connects simple devices to the Internet and manages communication between end-node devices and network gateways. Use of LoRaWAN in industrial spaces and smart cities is growing thanks to it is low cost, long-range and bi-directional communication, with very low power consumption.

### 2.1. LoRa

LoRa is the physical layer developed by Semtech [3], enabling the LoRaWAN end-device to achieve long-range communication through radio frequency modulation. LoRa modulation is based on chirp spread spectrum (CSS) [4]. It offers immunity to multi-path and tolerate small frequency offset caused by Doppler effect, making LoRa ideal for communication in urban environments. A chirp is characterized by a frequency excursion all over the bandwidth. The base chirp, or up-chirp, is characterized by linear increasing frequency, starting from the value $f_{min}$ and ending with a value $f_{max}$. Instead the chirp that starts with frequency $f_{max}$ and ends with $f_{min}$ is called down-chirp. For any digital bit sequence the LoRa device will produce a time shifted chirp to the respect of the base-chirp. In the CSS modulation the number of different symbols/chirps depends on the Spreading Factor (*SF*), $N_{Symb}=2^{SF}$. The value of the *SF* is also equal to the number of encoded bits per symbol. For example a modulated signal with *SF* = 7 can produce 128 distinguishable symbols each of one encoding a different sequence of 7 bits. Symbols are organized in frames (also named packets in the following). The frame structure is reported in Section 2.3. Packets with different spreading factors are orthogonal, appearing as a noise to each other. Therefore, two packets that arrive at the same time on the same receive channel at different spreading factors will not collide and, both will be demodulated by the gateway. However, two packets with the same spreading factor arriving at the same time on the same channel result in a collision. According to LoRa specification the *SF* may assume 6 different values, from 7 to 12. The bit rate $R_{b}$ depends on the *SF*. Given a bandwidth BW and a coding rate CR, the $R_{b}$ is given by the following expression:

(1) $$R_{b}=SF\cdot \frac{BW}{2^{SF}}\frac{4}{4+CR}bit/s$$

The higher the *SF* the lower the bit rate. If the same message is transmitted with two consecutive values spreading factors, e.g., 7 and 8, the time needed to transmit the message roughly doubles. The LoRa modulation characteristic vary for each region. In Europe, there are channels with bandwidth of 125 kHz and 250 kHz. LoRa transmits in the ISM band that in Europe is set in the frequency range 863–870 MHZ.

### 2.2. LoRaWAN

LoRaWAN is an open networking protocol that delivers secure bidirectional communication standardized and maintained by the LoRa Alliance [5,6].

**Figure 1:**
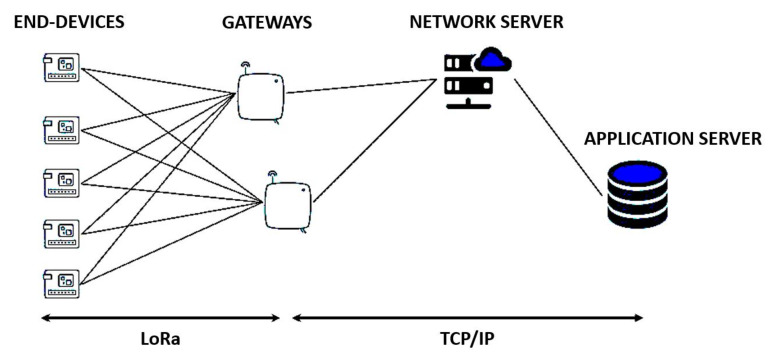
Simple representation of LoRaWAN and all its principal components.

The LoRaWAN architecture is deployed in a star-of-stars topology and it is composed by four principals components: End-devices (ED), Gateways (GW), a Network Server (NS), and application servers, as schematized in Figure 1. Gateways relay messages between end-devices and a central network server that forwards the message to or from the application sever. The gateways are connected to the NS via standard IP connections and act as a transparent bridge, simply converting radio frequency packets to IP packets and vice versa. The wireless communication takes advantage of the long range characteristics of the LoRa physical layer, allowing a single-hop link between the end-device and one or many gateways.

The LoRaWAN network server manages the entire network, dynamically controls the network parameters to adapt the system to every changing conditions. The NS carries on the over the air activation OTAA, started by the end-devices. This is responsible for the authenticity of the end-devices connected to the network, assigning to each of them a temporary unique identifier, the so called Device Address (DevAddr), and generating a 128-bit AES key in order to establish encrypted communication with the EDs. The NS schedules the acknowledgment and forwards it through the optimal gateway, which is evaluated on the Received Signal Strength Indicator RSSI value of the uplink packet. The NS dynamically manages the transmission parameters of the end-devices with the Adaptive Data Rate (ADR) algorithm. The transmission power and *SF* of the transmitted packet change in order to be adapted to the network load and maximize the throughput. Moreover, the NS is also responsible to perform packet de-duplication: a LoRa packet sent by an end-device could be received by multiple gateways and, therefore, the NS will receive multiple copies of the same packet. The NS should consider and/or forward to the application server only one copy of the packet.

LoRaWAN Gateway receives LoRa packets from any devices in the radio range and forwards these data packets to the NS, connected through IP backbone. Each ED can be served by multiple gateways in the radio frequency range. There is no fixed association between an end-device and the gateway. In this way, the packet error rate is reduced, since there is a higher probability that at least one gateway receives the packet, and significantly reduces the battery consumption for end-devices that are mobile. LoRaWAN gateway operates only in the physical layer forwarding the packets to the network server through an IP based Wi-Fi, Ethernet or Cellular backhaul. They only check the data integrity of each incoming LoRa packet. If the CRC status is incorrect the packet will be dropped. If correct the gateway will forward it to the NS, together with some metadata, such as the *RSSI* of the packet at the receiver. For the downlink packet, the gateway executes transmission requests coming from the network server, without any interpretation of the payload. It is up to the NS to select the appropriate gateway with which to transmit the downlink packet.

End devices are sensors or actuators that wirelessly communicate to a LoRaWAN through a gateway, using LoRa radio frequency modulation. They are autonomous battery operator devices. The LoRaWAN standard defines three classes of end-devices, Classes A–C. All such devices must support all operational modes of the lowest class. In other words, all the devices must be able to operate as Class A devices, Class B devices must support both Class A and Class B modes, and Class C devices must support all three modes of operation. The distinction between three Class devices have to do with how the device communicates with the network. In Class A devices, in order to establish bidirectional communication, each uplink transmission is followed by two short receiving windows during which the end-device listens for possible downlink traffic. It is the end-device to trigger the downlink communication. The two receiving windows start at 1 s and 2 s, respectively, after the end of the uplink transmission. Class B devices open other receiving windows at scheduled times, in order to increase the downlink capability. The Gateway transmits beacons to synchronize downlink communication between network server and Class B end-devices. Class C devices are all the time available to receive downlink communication, except when they transmit. Class A devices consume less power, since most of the time they are asleep. Instead, Class C are the ones with highest power consumption, since they are always active.

Before starting to exchange information in a LoRaWAN, each end-device has to be personalized and activated. The activation of an end-device can be completed in two ways: via Over-The-Air Activation (OTAA) or via Activation By Personalization (ABP).

To join the network through OTAA, an end-device needs to perform a join procedure before starting to exchange information with the network server. A very important point is that an end-device needs to perform a new join procedure each time it looses the session context information.

Before starting the join procedure the end-device has to be personalized with the following information:

- DevEUI: It is a global end-device ID in IEEE EUI64 address space that uniquely identifies the ED. DevEUI is the recommended unique device identifier by Network Servers, whatever activation procedure is used, to identify a device roaming across networks;
- JoinEUI: The JoinEUI is a global application ID in IEEE EUI64 address space that uniquely identifies the Join Server that is able to assist in the processing of the Join procedure and the session keys derivation;
- NwkKey and AppKey: They are AES-128 root keys specific to the end-device that are assigned to the ED during fabrication.

**Figure 2:**
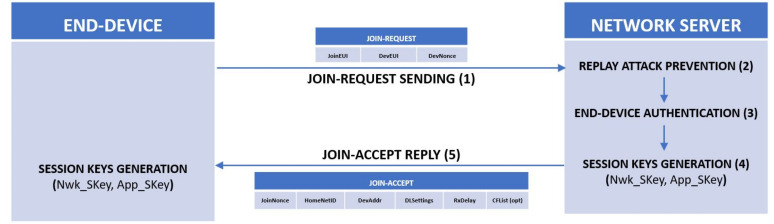
Over-The-Air Activation (OTAA) procedure flow graph.

The OTAA procedure can be resumed in five steps and graphically represented as in the scheme in Figure 2.

**Figure 3:**
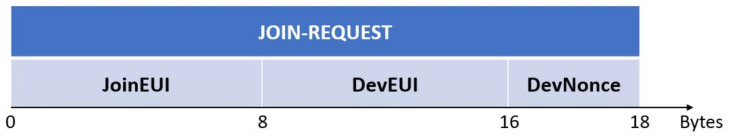
Join request message structure.

The join procedure is always initiated by the end-device by sending a join request message (in Figure 3) that is a message that contains the JoinEUI and DevEUI of the end-device followed by a nonce of 2 octets (DevNonce).

When the network server receives a join request, it performs the replay attack prevention process based on the validity of the DevNonce that needs to be such that a pair (JoinEUI, DevNonce) is a unique pair. If the join request is a valid one, the server performs the authentication of the end-device and if the ED passes the authentication, the NS generates the keys Nwk_SKey and an App_SKey.

**Figure 4:**
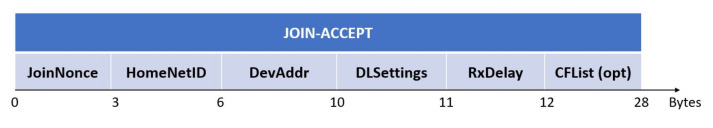
Join response message structure.

Once all the security checks are completed, the end-device is permitted to join the network, so the network server will respond with join-accept message, in Figure 4.

The JoinNonce is a device specific unique counter value provided by the Join Server and used by the end-device to derive the session keys FNwkSIntKey, SNwkSIntKey, NwkSEncKey, and AppSKey. JoinNonce is incremented with every Join-accept message. When receiving a join-response the end-device verifies that is a valid message by looking at the JoinNonce and if it is a valid one it stores the DevAddr in itself.

The DevAddr is a 32-bit ephemeral device address, that will identify the end-device during a session. When an end-device close or lost its session connection, its DevAddr becomes free and it will be reassigned another end-device, and if an end-device re-join the network a new DevAddr is assigned. Each end-device uses its DevAddr to derive the keys it has to use to send messages through the network.

Activation by penalization (ABP) directly ties an end-device to a specific network by-passing the join request–join accept procedure. In this case, the end-device already contains all what it needs to derive the keys to starting send messages. In other words, the end-device is equipped with the required information for participating in a specific LoRa network as soon as it is started.

### 2.3. LoRa Physical Packet Structure

In LoRa, there are two types of packets: downlink and uplink packets. Uplink are sent by end-devices to the NS relayed by one or many gateways. Uplink packets use the LoRa radio packet explicit mode in which the LoRa physical header (PHDR) plus a header CRC (PHDR_CRC) are included. The integrity of the payload is protected by a CRC.

Each downlink message is sent by the Network Server to only one end-device and is relayed by a single gateway. Downlink messages use the radio packet explicit mode in which the LoRa physical header (PHDR) and a header CRC (PHDR_CRC) are included.

**Figure 5:**
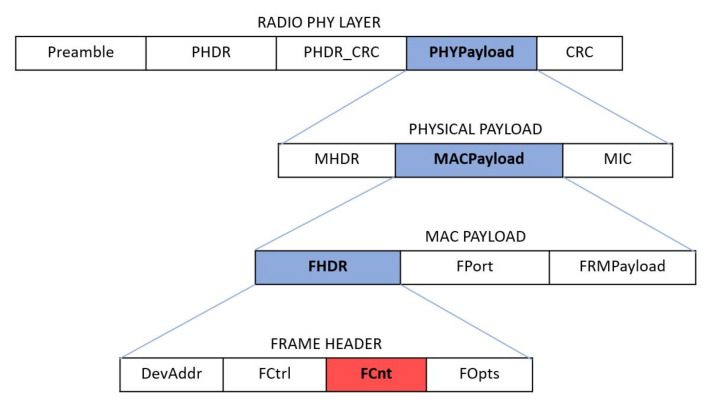
Structure of LoRa packet represented in a hierarchical schema. The CRC field in the radio physical layer is present only in uplink packets.

Both uplink and downlink messages carry a physical payload that contains all the information needed to forward the message through the network. The physical payload is composed by a single-octet MAC header (MHDR), followed by a MAC payload, and ending with a 4-octet message integrity code (MIC). The structure of a LoRaWAN frame is shown in Figure 5.

Inside the MAC payload there is the frame header (FHDR) that is the header of the packet at data link layer. The frame header is composed by device address (DevAddr) of 4 bytes, a frame control octet (FCtrl) of 1 byte, a 2-bytes frame counter (FCnt), and up to 15 bytes of frame options (FOpts) used to transport MAC commands.

The FCnt is a kind of counter that allows the devices to keep track of the number of data frames sent uplink to the Network Server (FCntUp), and sent downlink from the Network Server to the device (FCntDown). Whenever an OTAA end-device successfully process a join procedure their frame counters is reset to 0 while the ABP devices have their FCnt initialized to 0 at fabrication ant it must never be reset to 0.

### 2.4. LoRa Applications and Related Research

By using sensor devices with LoRa chipsets having a LoRaWAN connection, we can accommodate a vast range of IoT applications. Thanks to its flexibility, low cost, and robust modulation, the LoRa technology can be used in many different cases, such as smart agriculture, smart cities, industrial IoT (IIoT), smart environment, smart homes and buildings, smart utilities and metering, and smart supply chain and logistics [7].

One big challenge is to use LoRaWAN devices to build the next generation cities. In order to build a smart city there are at least four fields where LoRaWAN sensors may be employed: water and energy management, smart homes, indoor/outdoor asset tracking, and smart lighting [8].

To improve the water management in a city LoRaWAN sensors may be deployed in the existing water infrastructure to rapidly detect anomalies (e.g., water leaks) and reduce the waste of water. Since installation, the city of Lyon, France has identified and repaired 1200 water leaks, achieved an 8% increase in water network efficiency and saves an average of 1 million cubic meters of water annually [8].

On the other side, using LoRaWAN in a smart home allows to connect all the devices of the home, indoor and outdoor ones, using one network. We can think to a house with a garden, in this case a Wi-Fi network does not guarantee to connect all the devices of the home. By using LoRaWAN-enabled devices, we have a higher probability of accomplishing this, ensuring a smart home ecosystem with the highest degree of interoperability. To achieve the asset tracking goal the idea is to equip vehicles, products and trucks with LoRaWAN sensors in order to automatically locate, track, and monitor physical assets. The LoRa Edge [9] platform delivers one of the IoT industry’s lowest power and most affordable geolocation capabilities than traditional GPS technologies. The Yabby Edge features advanced Cloud-based location calculations, significantly reducing power consumption and extending battery life for up to 12 years. Location data can be easily forwarded to any customer platform or system for simple integration and device settings can be configured to fit any tracking application.

Moreover, LoRaWAN is already used in some cities to monitoring the resource consumption, such as light, gas, and water. OrionM2M is one of the first Kazakstani developers and manufacturers of wireless data transmission systems to integrate Semtech’s LoRaWAN devices and the LoRaWAN standard into its smart metering solutions to monitor water, gas, and electricity usage data, as well as smart lighting solutions to deliver operations management and service reliability, with up to 30% reduction in technical losses.

OrionM2M also exploits LoRaWAN to monitor COVID-19 storage and transportation in the last two years [10]. The monitoring system is based on six main points: (i) real-time monitoring of refrigeration chamber temperature parameters, (ii) automatic notification when temperature changes above the set threshold, (iii) logging of events both at the device level with a deep archive and in the system itself, (iv) analytics throughout the network for the required period with the formation and unloading of reports, (v) accounting for operating time and downtime of refrigeration equipment, and (vi) locations map of refrigeration chambers. The temperature and the humidity control is performed 24 h per 7 days and the detected values are stored hourly in a cloud archive. In case of anomalies a notification is sent to the distributor. There are some advantages in using LoRaWAN technologies in this case, the most relevant are the high reliability and availability of server applications backup, short terms of implementation and return of investment, scalability of the system and the ease to add and connect new devices to the network.

## 3. LoED: LoRaWAN Dataset

The analysis of the behaviour of LoRaWAN has been of carried out in many papers. Some of them leverage powerful simulation tools to observe the performance under different parameter settings [11] while others analyse real datasets by also employing classification methods to represent the behaviour [12].

In this work, we operate under the second approach. To this aim we used, as case study, the dataset “LoRaWAN at the Edge Dataset” (LoED), an open dataset of LoRaWAN packets collected at gateways during a period of time of 4 months, generated by smart city application and research deployment [2]. We selected the LoED dataset since, after a deep analysis of the literature it is one of the largest (1,263,001 entries) and most complete. This unique dataset contains traffic generated by several distinct IoT applications. Indeed, packet collection was completed at gateway-level, passively listening for LoRa frames in the air. This means that all packets observed by the gateways were captured, regardless of what device generated them or which application they belong to. The traffic was collected by 9 gateways in London, covering different city areas. Five gateways were deployed outdoors, on the rooftops of buildings, with clear line of sight to the devices. Four indoor gateways instead had limited line of sight, one of them was placed on the ground floor with no line of sight to any devices. The collected packets use the explicit header mode that allows to extract metadata information, such as:

- *time*: Time at which the packet was received by the gateway;
- *physical payload*: Raw payload contained in the received packet;
- *gateway*: Identifier of the gateway that has received the packet;
- *crc status*: Physical layer CRC;
- *frequency*: Transmission frequency;
- *Spreading Factor*: Transmission *SF* of the packet;
- *bandwidth*: Bandwidth used by the received packet;
- *code-rate*: LoRa coding-rate of the packet;
- *RSSI*: Sampled *RSSI* value of packet reception;
- *SNR*: Sampled SNR value of packet reception;
- *device-address*: Device-address of the device that has sent the packet;
- *mtype*: mtype bit fields of the packet;
- *fcnt*: Counter value of the packet;
- *fport*: Port of the packet.

The gateway numbers are hexadecimal string of 16 characters; for sake of simplicity we assign to each gateway an ID in the form of GWX where *X* is a number from 1 to 9. The matching of the IDs and the gateway numbers is in Table 1.

During the acquisition time were collected 11,263,001 packets from devices presenting 145,023 different device addresses.

We remark that the methodology here proposed can be applied to any other dataset if sufficiently rich. We suggest that experiments can be also carried out by deploying LoRaWAN gateways and using the The Things Network NS [13] to collect the relevant data and apply the proposed approach to test and measure the network behaviour.

## 4. LoRaWAN Analysis

In this section, we analyse several aspects of the LoRaWAN protocol. To this aim, we divided our study in six blocks, each focused on one specific behaviour of the network:

1. Gateway radio indicators;
2. Packets loss;
3. Device–Gateway interaction;
4. End-Device addressing and activation;
5. Duty-Cycle enforcement;
6. Performance of De-duplication procedure.

Let us first discuss what the dataset contains in terms of number of packets and types of packets. LoRaWAN foresees different “types” of packets, e.g., packets requiring an acknowledgment, join request messages, etc. The information characterizing the type of a packet is encoded with three bits in the field *mtype* in the MAC Header, which is sent as clear-text with the packet and it is reported inside the dataset. All the possible *mtype* values with their description is reported in Table 2.

To derive an initial characterization of the traffic in the dataset, we counted the total number of packets per *mtype*.

As described in Section 2.2, LoRaWAN protocol foresees the reception of duplicate copies of the same packet. In the dataset, such duplicate copies are included, therefore we counted the number of packets in two different ways, with and without duplicate copies.

Table 3 reports the total number of the packets contained in the dataset and the number of packets after removing the duplicate ones. In the dataset there are 11,263,001 collected packets and 8,266,868 distinct packets. The packets identified as “Unknown” are those packets which are captured by the LoRa antennas on the gateways but do not follow the LoRaWAN protocol. Most of them have an empty PHYPayload, and could be beacon packets for some proprietary systems over LoRa. We consider all these packets as distinct since we cannot determine whether a duplicate reception takes place.

In the following sub-sections we first introduce the features we want to analyse, then we present our strategy to carry out the specific analysis and the results obtained applying the functions of our library.

### 4.1. Gateway Radio Indicators

The goal of the first analysis is to analyse the behaviour of the two radio indicators Received Signal Strength Indicator (*RSSI*) and Spreading Factor (*SF*). By studying the behaviour of these parameters, we can understand the performance as observed at the gateway level. The *RSSI* represents, in dBm, the received signal power. This value can be used as a measurement of the quality of the signal received by the gateway.

The *RSSI* reported in LoED is the one measured at the gateway and it represents the strength of the signal received by the gateway for the uplink messages.

The Spreading Factor (*SF*) controls the chirp rate of an end-device in a LoRaWAN, and, thus, controls the speed of data transmission. The *SF* can be managed by the NS dynamically with the Adaptive Data Rate (ADR) protocol together with the ED, or it can be arbitrarily set by the ED. Lower spreading factors mean faster chirps and, therefore, a higher data transmission rate. For every increase in spreading factor, the chirp sweep rate is halved and so also the data transmission rate is halved. Moreover, the higher the *SF*, the higher the probability of a successful reception of a packet by a gateway.

Table 4 shows the *SF* with the resulting bit rate. In order to analyse the behaviour of *RSSI* and *SF* values of the gateway in LoED we split the packets by the receiving gateway and for each sub-set we store the values of *RSSI* and *SF* for each packet.

**Figure 6:**
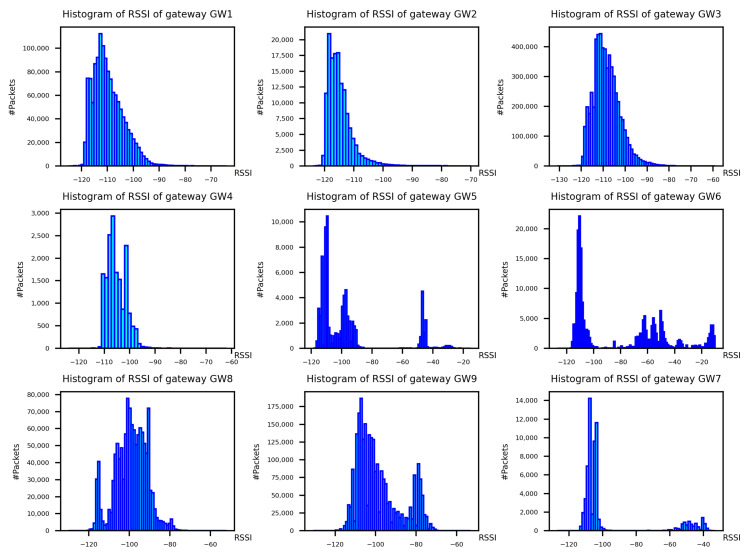
Histogram of *RSSI* per gateway.

**Figure 7:**
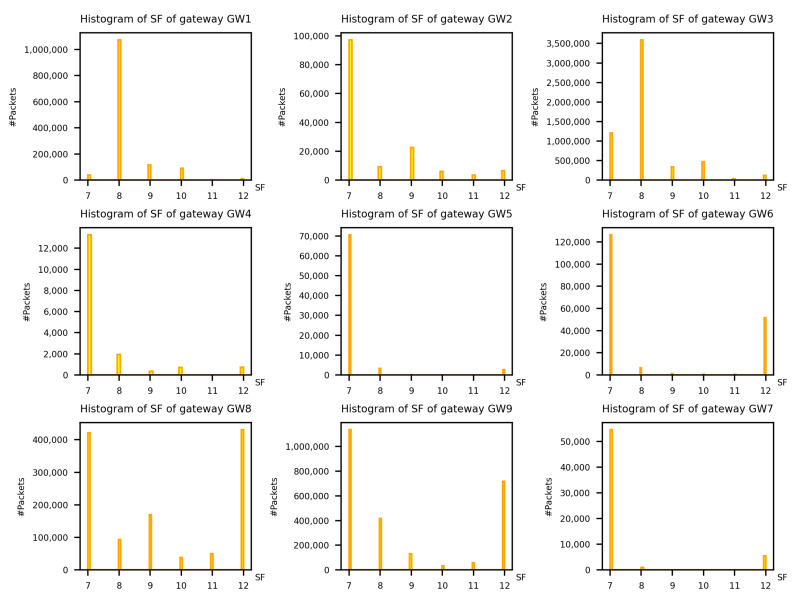
Histogram of spreading factor per gateway.

In Figure 6 and Figure 7, the histograms of *RSSI* and *SF* per gateway are reported, respectively. Both values are measured at the gateway level.

By looking at Figure 7, we can observe how the majority of the LoRa packets assume a low value of *SF*, around 7 or 8. This is due to achieve the maximum data rate, to use less network resources and to be as energy efficient as possible.

With gateways GW8 or GW9 we can observe a large component of packets assuming *SF* = 12. This observation is coherent with the geographical placement of such gateways. Indeed, they are placed on top of tall buildings with no obstacles around them, this enables the reception of packets sent by distant ED, which set their *SF* high in order for their packets to be received.

Regarding the plots in Figure 6, they shown how the *RSSI* behaviour is different for each gateway. The three gateways GW1, GW2, and GW3, that correspond to the three plots of Figure 6 have a dense and well defined *RSSI* distribution with an average of around −110 dBm and with an exponentially decaying tail of higher *RSSI* values. The same reasoning could be applied to GW8 and GW9, although with a less well-defined distribution. All these gateways are deployed on top of buildings without having lots of obstacles. This causes the gateways to receive a high number of LoRa packets and to have a well-defined distribution of *RSSI* values.

Gateways GW4, GW5, and GW6 are smaller gateways deployed indoors inside a university building. They do not collect a high number of packets and we can observe two distinct peaks in their *RSSI* distribution. One peak is located at −110 dBm and the other around −50 dBm. The first peak is due to packets generated by devices outside or distant from the gateways. On the other hand, the latter is probably generated by devices which are inside the same building and within close proximity to the gateways.

### 4.2. Packets Loss

In this section we focus on the packet loss in a LoRaWAN. Packet loss in this kind of network can be caused by a variety of factors. In particular, research efforts have been carried out to derive a model for packet collision at the LoRa physical layer [14,15].

The majority of the LoRaWAN traffic is “Unconfirmed”, i.e., without requiring an acknowledgment, as it is reported in Table 3. The unconfirmed traffic demands less resources from the LoRaWAN and it is more suitable for devices having a limited energy supply. However, the NS is unaware of lost unconfirmed uplinks, since acknowledgments and/or retransmissions are not required.

Our goal is to compute a distribution of the packets lost in the transmission. We split the dataset by DevAddress. For each device, we consider the FCnt counter in the frame header (highlighted in red in Figure 5. FCnt is a 16-bit counter which increases its value by one for each uplink packet sent by an ED. The FCnt is a value reported in the header of the packet and it is sent as plain-text, i.e., one can analyse the ratio of lost packets even without having the decryption keys.

**Figure 8:**
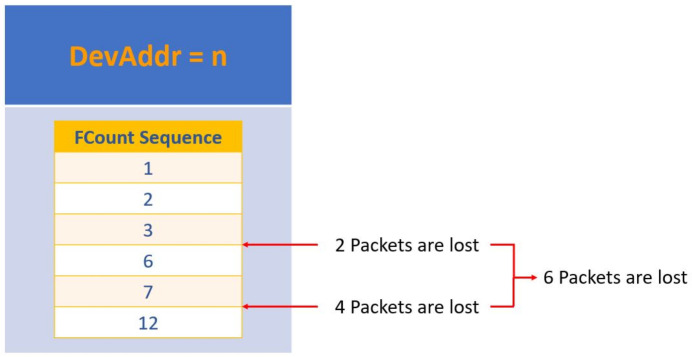
Scheme of the adopted strategy to compute the number of lost packets for a given DevAddr.

If any packet is lost, then the observed values of FCnt are continuous. However, if some packet is lost, then we will observe some missing values of FCnt. Precisely, if we observe a packet with FCnt = *n* and successively we observe a packet from the same ED with FCnt = *m*, the number of lost packets is m−n−1. This strategy is schematized in Figure 8.

**Figure 9:**
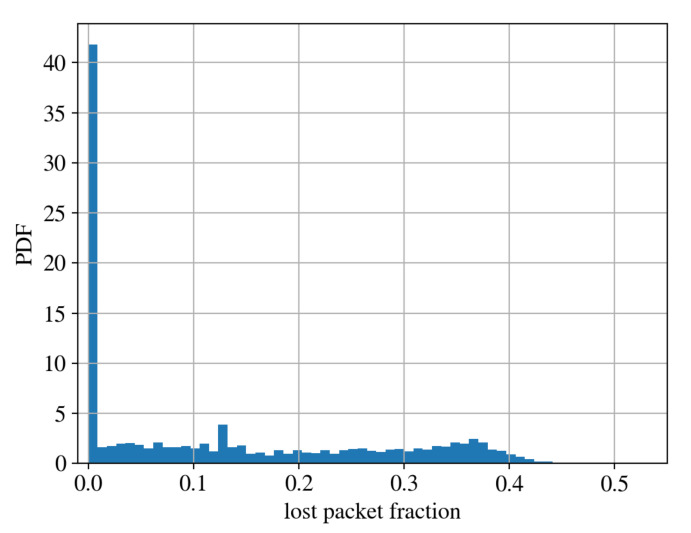
PDF distribution of lost packets. To compute it, we isolated the packets of each device in the network. Then, we computed the ratio of lost packets for each device. The PDF in figure is the distribution of those ratios.

**Figure 10:**
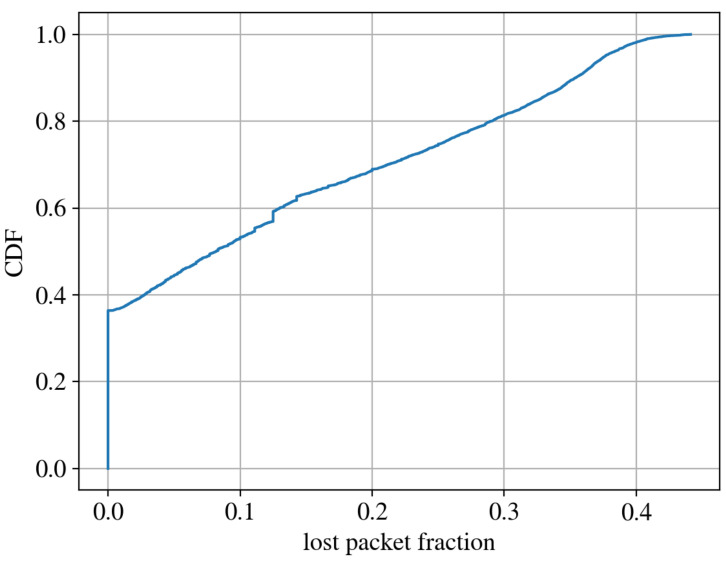
Plot of CDF of devices in function of percentage of packet loss.

In order to compute the distribution of the lost packets, we first isolate the packets of all the devices in the network. For each device, we then compute the ratio of the lost packets using the strategy described previously. By computing the PDF distribution of such values, we obtain the result shown in Figure 9. In Figure 10, the same distribution is reported as a CDF. To increase the robustness of the analysis, we considered only packets belonging to those devices which transmit regularly to the network. By looking at Figure 9, we can observe that the majority of the devices loses a small fraction of packets, while others lose up to 50% of packets.

To perform this analysis, we considered $N_{C}$ = 3,374,952 uplink packets correctly received and we computed $N_{L}$ = 1,333,398 lost packets. The cumulative fraction of lost packets in the whole LoRaWAN is, therefore:

$$\mathrm{lost}=\frac{N_{L}}{N_{C}+N_{L}}=0.283$$

### 4.3. Device-Gateway Interaction

The goal of this analysis is to investigate the number of devices handled by each gateway. Since we do not know the unique identifier of a device (DevEUI), we can count the number of “temporary” devices, which are differentiated by DevAddress field encoded as clear-text in the header.

**Figure 11:**
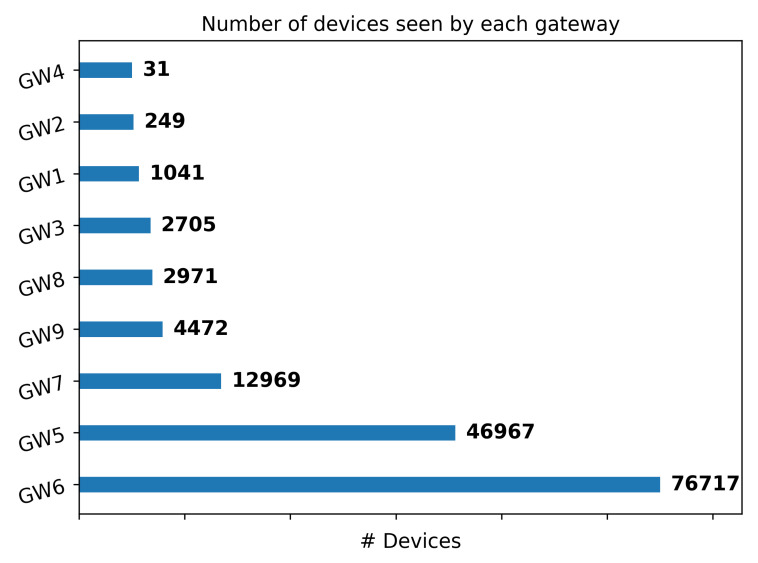
Histogram representing the number of devices managed by each gateway in the network.

We have parsed the dataset and group the packets by gateway. Then we have counted the number of distinct device-address seen for each gateway. We plot the obtained results in Figure 11.

The histogram reported in Figure 11 represents the number of distinct device-address seen by each gateway. We observe that the two gateways that have seen the highest number of distinct DevAddress, GW6, and GW5, are the ones which were up and running by the longest time period, of 552 and 573 days, respectively. However, the same gateways are placed in an indoor environment, which is not optimal for receiving LoRa packets. Indeed, they have managed only 186,592 and 76,706 packets, respectively.

On the other hand, GW3 manages packets generated by only 2705 distinct DevAddress, but it is the one that handles the highest number of 5,757,575 packets in a relatively short period of activity of 56 days.

### 4.4. End-Device Addressing and Activation

In this section, we investigate the behaviour of the Over-the-Air Activation (OTAA) by looking at the device addresses and the real identifiers of the devices.

The OTAA activation method foresees an exchange of join request/join accept messages between an ED and the NS. During this procedure, several network parameters could be set by the NS, including the DevAddress, a public ephemeral identifier for the ED, which will be sent as plaintext in every Confirmed/Unconfirmed Data Up packet.

During its lifetime, an OTAA device, could assume several distinct values of a DevAddr. However, it has one and only DevEUI, which is a unique identifier for a LoRaWAN device. The DevEUI is assigned by the manufacturer in a similar way of a MAC address, containing information about the producer and the unique identifier for the ED.

The DevEUI is not used in Confirmed/Unconfirmed traffic for several reasons including privacy [16] and, in general, it is not reported in public datasets. However, the DevEUI is sent as plaintext in a join request message. This opens us a possibility to fetch the DevEUI of the devices that performed an OTAA procedure in the LoED dataset.

**Figure 12:**
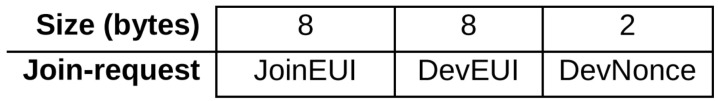
Structure of the PHYPayload of a join request message.

As stated earlier, the entries of LoED dataset do not have the DevEUI of the end-device that has sent the packet but we can retrieve the DevEUI from the payload of the join request messages. In order to retrieve the DevEUIs, we filter all the join request messages that we identify by looking at the *mtype* field, that in the case of join request is equal to 000. Once we have identified a join request we consider its payload, and we extract a substring of 8 bytes, from the 8th to the 15th, as shown in Figure 12.

By following this strategy, we counted the number $N_{DevEUI}$ of distinct DevEUI in the dataset $N_{DevEUI}=1481$. $N_{DevEUI}$ represent the number of distinct devices which are present in the dataset and have gone through an OTAA procedure at least once. The number of distinct DevAddr is instead $N_{DevAddr}=49,876$. We can compute the ratio between $N_{DevAddr}$ and $N_{Dev}$:

$$\frac{N_{DevAddr}}{N_{Dev}}≃33.68$$

This ratio represents, on average, the number of DevAddress assumed by a single ED.

We also have analysed the number of join request, rejoin request and join accept. We could easily filter them by the *mtype* field, since these message types have *mtype*, respectively, set to 000, 001, and 110. Without considering the duplicate messages we have the following:

- Number of join request: $N_{JoinReq}=170,476$;
- Rejoin request: $N_{RejoinReq}=26,561$;
- Join accept: $N_{JoinAcc}=20,660$.

We then count the average number of join procedures per device. To perform this counting, we have consider a completed join procedure per join accept. By the LoRaWAN standard, the response of a rejoin request is not always a join accept but it could be a normal downlink message with, for example, MAC commands setting network parameters. For that reason, we consider also a completed join procedure per rejoin request. Finally, to compute the average number of completed join procedures per device we apply the following relation:

$$\frac{N_{JoinAcc}+N_{RejoinReq}}{N_{Dev}}≃32$$

So we have, on average, 32 completed join procedures per device during the period of packet collection of the dataset. Furthermore, we have analysed the ratio of the join accept messages to the observed number of DevAddr.

$$\frac{N_{JoinAcc}}{N_{DevAddr}}=0.41$$

The ratio of 0.41 highlights the fact that in the network we have more DevAddr than the number of completed join procedure. The extra number of Dev Address could be due to ABP devices (which do not require a join procedure) and/or devices which have already performed a join procedure before the start of the packet collection.

### 4.5. Duty-Cycle Enforcement

**Figure 13:**
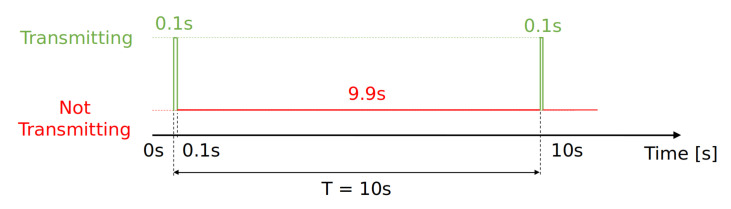
Schema representing behaviour of device respecting a duty-cycle of 1%.

The LoRaWAN standard foresees a regulation on the duty-cycle that all devices should enforce. In the case of LoRaWAN, the duty-cycle is the ratio of time during which a packet is actively sent on the air over the time of radio silence. An example of duty-cycle behavior is reported in Figure 13.

The duty-cycle limitations depends on several factors including the region of operation, on the channel in use or the type of packets sent. In the band EU868 the maximum duty-cycle attainable is 1.0%, i.e., a device can send packets on the air 1% of the time and should be silent 99% of the time. The time of operation of the radio depends on the Time-on-Air of a packet and ultimately on the LoRa radio parameters. For example, if a device sends a join request (23 bytes of payload) using *SF*10 and a 125 kHz channel with a final ToA of ≈500 ms, it needs to wait 99×500 = 49,500 ms before sending a new message.

We investigate whether the duty-cycle constraints are respected by the end-devices in the real LoRaWAN depicted by the LoED dataset. Since the LoRaWAN used to collect LoED dataset is based in London, we look LoRaWAN specification to see the constraint on the duty-cycle imposed in United Kingdom and it has to be <1%.

The strategy used to compute the duty-cycle for each device seen in the network follows essentially three steps:

1. Find the period of time of connection of the device to the network, computed as $T_{Dev}=T_{Dev}^{f}-T_{Dev}^{i}$ where $T_{Dev}^{i}$ is the first time in which the device is seen in the network and $T_{Dev}^{f}$ is the last time in which the device is seen in the network;
2. Compute the amount of time $T_{Dev}^{act}$ in which the device was active, we find this amount time by sum all the ToA of the packets sent by the device;
3. Compute the duty-cycle percentage as
  $$\%DutyCycle=\frac{T_{Dev}^{act}}{T_{Dev}}100.$$

The previous three steps are performed for each device address observed in the network. In order to calculate the ToA of a packet we applied the following formula:

$$ToA=\;\left(m_{ph}+\frac{8\times P}{4\times SF}\times \left(4+RDD\right)\right)\;\times \frac{2^{SF}}{BW}$$

In that relation we have:

- Preamble $m_{ph}=1$;
- RDD=1;
- Spreading factor of the packet SF;
- Length of the payload *P* of the packet;
- Bandwidth used to transmit the packet expressed in Hz.

By applying the strategy explained before we obtain the following plots.

**Figure 14:**
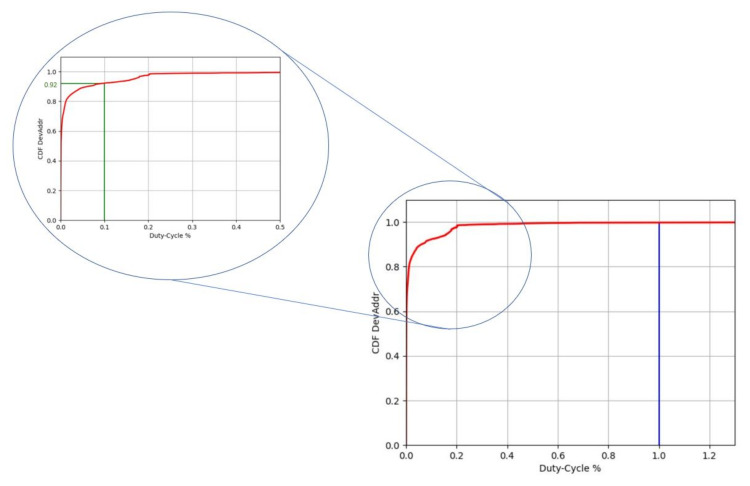
Plot of CDF of end-devices in the function of the percentage of the duty-cycle with zoom on duty-cycle percentage range [0, 0.5]. In the complete graph, the blue line represents the duty-cycle constraint of 1% while in the zoom the green line represents the duty-cycle of 0.1% and the correspondent CDF of 0.91.

In the bottom-right part of Figure 14 the plot of the CDF distribution of all the device over the percentage duty-cycle is reported. The constraint of 1% correspond to the vertical blue line and as we can see by looking at the plot, this constraint is respected by all the devices. In the zoomed section, of the plot there is a green line representing the other most common LoRa duty-cycle constraint of 0.1%. It is also specified the CDF value of 0.91 correspondent to the intersection between the vertical green line and the red curve. This means that the 91% of the devices maintain the duty-cycle lower than 0.1%.

### 4.6. Performance of De-Duplication Procedure

In a LoRaWAN, each time the NS sends a downlink packet it has to select the gateway that will forward the packet to the end-device, that operation is simple when it receives a non-duplicate uplink packet, in this case the NS will select the gateway from which it has received the uplink packet. However, in a multi-gateway network, a packet sent by a device could be received by more than one gateway. All the duplicate packets are then forwarded by the gateways to the NS. In this scenario, the NS has to operate a packet de-duplication, in which only one packet among the multiple copies is kept and forwarded to the Application Server [4]. Moreover, the gateway which received the de-duplicated packet, is the one selected to send a downlink packet to the device, if needed.

LoRaWAN specifications do not specify the procedure that the NS should follow to operate de-duplication and gateway selection. One possible criterion tho select one gateway is to choose the one that has forwarded the uplink packet with higher SNR [17] or the higher *RSSI*. In order to collect all the uplink packets, the NS opens a time window of duration $T_{D}$ when a new and unseen packet is observed. This window is called de-duplication window. In LoRaWAN specification there are no fixed values to assign to $T_{D}$, however some of the most known open source implementations of LoRaWAN NS [13,18] use the value $T_{D}=200$ ms.

To investigate the behaviour of duplicate packets, we analyse the packets as if we were a NS performing a deduplication procedure, carefully selecting the value of $T_{D}$.

**Figure 15:**
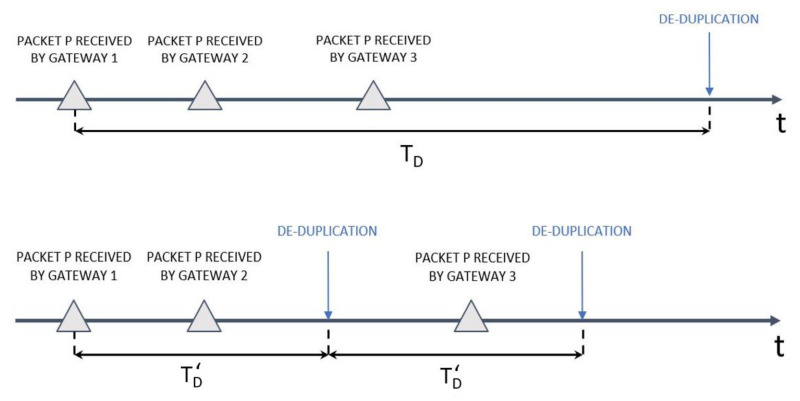
Scheme of the de-duplication behaviour in function of the time. In the upper scheme is represented only one de-duplication window $T_{D}$ while in the scheme below has two de-duplication windows of time $T_{D}^{\prime }$. At the end of each de-duplication window the network sever performs the ms.

Figure 15 represents two cases of de-duplication, in both cases there are three duplicates of the packet *P* but the two differ on the duration of the de-duplication window. In the upper scheme, $T_{D}$ is such that all the duplicates are taken into account for the de-duplication procedure, so in this case all the duplicates are deleted and only one response is sent. The inconvenient in this first case is that $T_{D}$ is too big, in fact the network server, after receiving packet *P* by gateway 3, waits for a long time before to perform the de-duplication even if there are no more duplicates.

In the second case, represented in Figure 15, there are two de-duplication windows of time $T_{D}^{\prime }$ and in this case two duplicates are considered in the the first de-duplication and the last arrived duplicate arrives in the second de-duplication window. Since the duplicates are divided in two de-duplication windows the network server will delete the duplicate in the first de-duplication window and consider the third duplicate as wrong packet because it has an already used frame counter. In this case, the network server may not wait enough to chose the best gateway to forward the response.

Our goal is to find a good trade-off for $T_{D}$ that will be used by the NS. First, we search for the duplicate packets and analyse the time $T_{F}$ where the first duplicate appears and the time $T_{L}$ where the last duplicate appears, for each group of duplicate frames. Then, we have used these values to compute the optimal de-duplication window duration for each duplicate packet p as follow:

$$T_{D}^{p}=T_{L}^{p}-T_{F}^{p}$$

After computing the optimal de-duplication window duration for each group of duplicates, we plot the distribution of the optimal de-duplication window time over the quantity of groups of duplicates.

**Figure 16:**
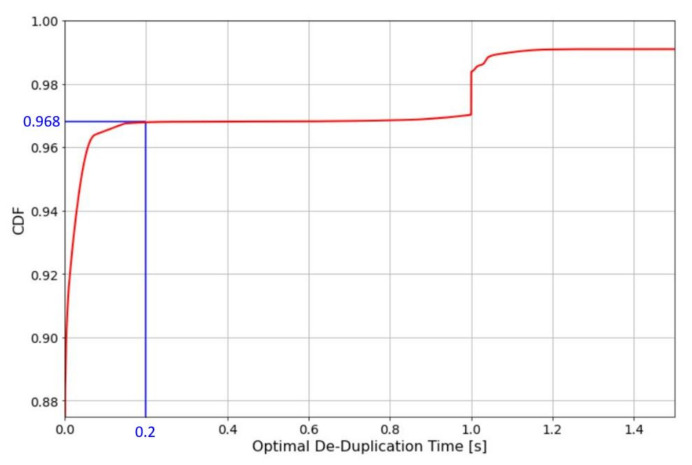
Plot of the distribution of the groups of duplicates over the optimal de-duplication time expressed in second.

Figure 16 reports that the results of our analysis show we can consider as a good de-duplication time the value that corresponds to the first elbow after which the distribution curve is flat, this value is 200 ms. By assigning $T_{D}=200$ ms we have a good trade-off between duplication elimination an network performances, in fact if $T_{D}=200$ ms it is not to high we will detect the 96.8% of duplicates.

## 5. Discussion

In this section, we give a brief discussion on the tests we have performed with the proposed methodology. The very first observation is that all the obtained results are coherent with the LoRaWAN specifications: the relationships among the different analyses confirm that the observed performance respect the required behaviour.

In the first analysis in Section 4.1 we have analysed the RSSI and *SF* of all the packets exchanged into the network. These parameters are all in the expected ranges, i.e., the RSSI is greater than −120 dBm and the *SF* in the range [7, 12]. As discussed in Section 4.1, the behaviour of the values of the RSSI and *SF* are coherent with the geographical position of the gateways. Although the majority of the packets are forwarded with *SF* 7 and *SF* 12 for GW8 and GW9, the RSSI is more variable. In fact, in the adaptive data rate mechanism, before changing the *SF* value, the LoRaWAN devices try to adapt the power to attain a given RSSI and maintain a good transmission quality.

In Section 4.2, we exploited the packet field FCount to count the number of lost packets per device. All the tested devices have a packet loss percentage lower than 45% while on average the packet loss percentage is 28.3%. We notice that it is fundamental to have this measure in order to face issues that may arise for some applications for which loosing 28.3% of the packets could be critical. However, this is part of the existing trade-off when we want to use a low power consumption network on one side and a reliable transmission on the other side.

Looking at the RSSI behaviour represented in Figure 6, it is clear that all the gateways try to set the transmission parameters in order to guarantee the best forwarding conditions. What we can also observe is (Section 4.3) the number of devices captured by the gateways, that is strictly related to the position of the gateway and its activity time. In fact, the gateway that managed more devices are the GW5 and GW6 that are also the ones which were up and running for the longest time period, 552 and 573 days, respectively. Looking at GW4 and GW7, they have the same activity time period, 15 and 17 days, respectively, but GW7 has managed 12,969 different devices while GW4 only 31. This because the GW4 is placed in a very bad position with a very constrained line of sight.

Another key function in a LoRaWAN is the join procedure, that is the procedure through which LoRaWAN end-devices can join the network. In Section 4.4, we firstly found the ratio between the number of DevAddr and number of distinct physical devices measured by counting the distinct DevEUI. This ratio is 33.68 which means that on average for each physical end-device there are 33.68 different DevAddrs. To confirm that, we also measure the ratio between the number of completed join procedures and number of physical devices obtaining 32 completed join procedures per device. The last interesting aspect we have noticed, by observing the join messages, is that the ratio between the number of join-accept packets and the number of physical devices is of about 0.41. That means that only 41% of the devices in the network join the network for the first time or through OTAA procedure. The remaining 59% was already in the network before starting collecting packets in the dataset or have joined the network through ABP.

One of the parameter imposed in the LoRaWAN protocol is the duty-cycle. In Section 4.5, we have measured the duty-cycle per device following the specific procedure performing the three steps detailed in Section 4.5. We have found that all the devices observed in the network respect the constraint of 1% duty-cycle.

The last properties we focused on is the de-duplication window time. This is the time that a NS spends waiting for duplicates packets. Since there are no clear specification on that time we have searched for an optimal de-duplication window duration in our case by collecting groups of duplicates and for each group by computing a suitable de-duplication time (Section 4.6). By plotting the optimal de-duplication windows duration (Figure 16) we have identified as a global optimal de-duplication windows duration 200 ms. We recall that by using the proposed methodology in another dataset the results may be different since they depend on the specific traffic managed in the network.

## 6. Conclusions

In this work, we dealt with Low-Power Wide-Area-Network and specifically with LoRaWAN. These networks use lightweight protocols to manage efficiently power-consumption, signal range coverage and data transmission in IoT frameworks. The goal is twofold: (i) to present into detail some methodologies (and to provide relevant software tools) able to capture the behaviour of these kind of network by analysing traffic traces stored in datasets; (ii) to test and perform the analysis of some data collected in a quite rich datasets identified in the LoED one.

That dataset is quite a large, contains 11,263,001 real world LoRaWAN packets and is collected from a wide network deployed in London counting nine different Gateways and more than 1481 distinct physical end-devices. This dataset is interesting not only for the dimension of the network from which the data are collected but also because the network is used for multiple applications and for a long period of time.

The result of the work is a data analysis allowing us to understand how a real LoRaWAN network works and in particular how all its components cooperate to reach the desired performance. More, we also checked if, in this case study, all the specifications are respected.

We have performed six different analyses. Each analysis focuses on a particular feature of LoRaWAN and for each of them we have explained the strategy used to carry it out and we have presented the obtained results.

As expected all the obtained results verify the LoRaWAN specification. By looking at the results we can observe how the gateway modifies the radio indicators such as Spreading Factor and *RSSI* in order to guarantee good performances at any time and at any conditions.

## Figures and Tables

**Table 1:** Gateway numbers to gateway IDs.

Gateway Number	Gateway ID
00000f0c210281c4	GW1
00000f0c22433141	GW2
00000f0c210721f2	GW3
00000f0c224331c4	GW4
00800000a0001914	GW5
00800000a0001793	GW6
00800000a0001794	GW7
7276ff002e062804	GW8
0000024b0b031c97	GW9

**Table 2:** Table reporting all *mtype* fields foreseen by LoRaWAN with their related message type.

mtype	Message Type
000	Join request
001	Join accept
110	Rejoin request
010	Unconfirmed data-up
100	Confirmed data-up
011	Unconfirmed data-down
101	Confirmed data-down
111	Proprietary

**Table 3:** Number of packets contained in LoED dataset grouped by message type. In the centre column it is reported the total number of packets including the duplicates, while on the right it is reported the number without duplicates.

Message Type	Total Number of Packets	Number of Distinct Packets
Join request	246,272	170,476
Rejoin request	27,576	26,561
Join-Accept	20,971	20,660
Confirmed data-up	664,474	441,742
Unconfirmed data-up	7,441,505	4,776,723
Confirmed data-down	63,216	32,537
Unconfirmed data-down	24,140	23,989
Proprietary	30,206	29,539
Unknown	2,744,641	2,744,641
**Total**	**11,263,001**	**8,266,868**

**Table 4:** Table reporting spreading factor values with relative bit rate. [4]. In this table we use the 125kHz bandwidth channel.

Spreading Factor	Bit Rate [bps]
*SF*12	150
*SF*11	260
*SF*10	580
*SF*9	1360
*SF*8	2740
*SF*7	4840

## Data Availability

The dataset LoED is publicly available here https://zenodo.org/record/4121430#.YjMRoX_MJhE (accessed on 30 January 2022).

## Abbreviations

The following abbreviations are used in this manuscript:

ABP	Activation By Personalization
ADR	Adaptive Data Rate
BW	Bandwidth
CR	Coding Rate
CSS	Chirp Spread Spectrum
DevAddr	Device Address
DevEUI	Device Extended Unique Identifier
DevNonce	Device Nonce
ED	End Device
FCnt	Frame Counter
FCntDown	Frame Counter Downlink
FCntUp	Frame Counter Uplink
FCtrl	Frame Control
FHDR	Frame Header
FOpts	Frame Options
ISM	Industrial Scientific and Medical
LoED	LoRaWAN at the Edge Dataset
LPWAN	Low-Power Wide-Area-Network
MHDR	MAC header
MIC	Message Integrity Code
NS	Network Server
OTAA	Over-The-Air Activation
PHDR	Physical Header
*RSSI*	Received Signal Strength Indicator
*SF*	Spreading Factor
